# Supplementary material for: Environmental Impacts of High-Quality Brazilian Beef Production: A Comparative Life Cycle Assessment of Premium and Super-Premium Beef
Source: Animals (Basel). 2023 Nov 20;13(22):3578. doi: 10.3390/ani13223578 (PMC10668795; doi:10.3390/ani13223578)
Supplement: Supplementary file 1 [file animals-13-03578-s001.zip › animals-2629418-supplementary.pdf]

animals

Supplementary Material

Environmental Impacts of High-Quality Brazilian Beef Production: A Comparative Life Cycle Assessment of Premium and Super-Premium Beef

Henrique Biasotto Morais[1,*], Luis Artur Loyola Chardulo[1,2], Welder Angelo Baldassini[1,2], Isabella Cristina de Castro Lippi[2], Gabriela Belinassi Orsi[2] and Clandio Favarini Ruviaro[3]

[1] UNESP – FCAV, Jaboticabal-SP, Brazil
[2] UNESP – FMVZ, Botucatu-SP, Brazil
[3] UFGD - FCA, Dourados-MS, Brazil
* Correspondence: biasotto.morais@unesp.br; Tel.: +55(19)991147207

Equation		Emission fators and others<br>Constants		
Enteric methane emissions				
10.3	Maintenance (NEm)	Cfi		
			adult female	
Chapter 10 IPCC 2006	NEm = Cfi • (Weight)^0.75	0.322	non-lactating	
		0.37	adult male	
10.6	Growth (NEg)	C		
Chapter 10 IPCC 2006	NEg = 22,02*(BW/C*MW)^0,75 * WG^1,097	0.8	female	
		1.0	male	
10.14	Ratio of net energy available in diet for			
Chapter 10 IPCC 2006	maintenance to digestible energy consumed (REM)			
	REm = 1,123-(4,092*10^-3*DE%)+(1,126*10^-5*($DE\%^2$))-25,4/DE%			
10.15	Ratio of net energy available for growth in			
Chapter 10 IPCC 2006	a diet to digestible energy consumed (REG)			
	REg = 1,164-(5,16*10^-3*DE%)+(1,308*10^-5*DE%^2)-(37,4/DE%)			
10.16	Gross Energy (GE)			
Chapter 10 IPCC 2006	GE = (NEm/REM + NEg/REG)/(DE%/100)			
10.17	Dry matter intake: (DMI)			
Chapter 10 IPCC 2006	DMI = BW^0,75*(0,2444*NEma-0.0111*$NEma^2$-0,472)/NEma			
	NEma = REM x 18.45 x DE% / 100			
10.21	CH4 emission factors for enterc fermentation			
Chapter 10 IPCC 2006	from a livestock category: (EF)	Ym		
	EF = (GE*Ym*days)/55,65 MJ/kg CH4)	0.05		
manure management				
10.22	CH4 emissions from manure management, for a defined population			
Chapter 10 IPCC 2006	CH4Manure = EF(T)*N(T)/10^6			
10.23	annual CH4 emission factor for livestock category (EF)	MS(T,S,k)	MCFsk	Bo(T)
Chapter 10 IPCC 2006		0.99	1.5	0.1
10.24	volatile solid excretion per day on a dry-organic matter basis	UE		
Chapter 10 IPCC 2006	VS = GE*(1-DE/100)+(UE*GE)*(1-ASH/18.45)	0.04		
10.25	Direct N2O emissions from manure management	MS(T,S)	$EF^3(S)$	

Chapter 10 IPCC 2006		0.99	0.02

10.30	annual N excretion for livestock category	Nrate(T)
Chapter 10 IPCC 2006	Nex(T) = Nrate(T) * TAM(T)/1000 * days	0.36
